# Book Reviews

**Published:** 1900-06

**Authors:** 


### Book Reviews.

A Cyclopedia of Practical Medicine and Surgery. A Concise Reference Book, alphabetically arranged, of Medicine, Surgery, Obstetrics, Materia Medica, Therapeutics and the various specialties, with particular reference to diagnosis and treatment. Compiled under the editorial supervision of George M. Gould, M. D., and Walter L. Pyle, M. D. Philadelphia: P. Blakiston's Son & Co. 1900.

It is a great task to prepare a single volume that shall provide a cyclopedia of medicine and surgery. Moreover, it is one that has rarely been accomplished. The field is so large, the subjects are in great part so complex, and the difficulty of condensation without loss of substance is so great, that few men would dare undertake such a Herculean labor.

These editors, however, have accomplished the work they set themselves about in a most thorough manner; they have succeeded in incorporating in the book a vast number of short, pithy articles, all of which will prove useful, more useful oftentimes, perhaps, than the long discoursive articles in the larger systems. It appears to have been one of the aims of the editors to give proper consideration to those slighter ailments which are so often overlooked in the text-books, and a glance through its pages will show a large number of references that are not to be found elsewhere.

The book being a practical one, diagnosis and treatment have received particular attention. Many valuable formulæ have been included and a large amount of information presented in tabular form, all of which makes this volume a practical working book that will be constantly referred to in every-day practice. It is strongly bound in sheep and is handsomely printed.

TRANSACTIONS OF THE MISSISSIPPI VALLEY MEDICAL ASSOCIATION. Twenty-fifth Annual Session, held at Chicago, Ill., October 3, 4, 5 and 6, 1899. Volume I. Edited by HENRY E. TULEY, M. D., Secretary, Louisville, Ky. Octavo, pp. 474. Louisville, Ky.: The Courier-Journal Print. 1900.

This influential and important medical association takes a new departure this year in the publication of its proceedings. It met in Chicago, continued its sessions during four days, organised in two sections—medical and surgical—and listened to 47 papers, most of which were ably and amply discussed. It is most fitting that such work should be presented for inspection in book form. The Association has adopted this method of publication, and is to be congratulated thereupon.

Whatever may be said in favor of publishing such material journalistically, it will ever remain a fact that nothing is so satisfactory as a complete book with the whole proceedings open to inspection at a glance, in which, too, a particular group of subjects may be read easily at a short setting.

This volume is well arranged, handsomely printed and bound, and contains much valuable material. The publication committee consists of Dr. Henry E. Tuley, chairman; Dr. L. S. McMurtry and Dr. Dudley S. Reynolds. The book is edited by Dr. Tuley, and to say the work has been well done only feebly expresses a very apparent fact, which may be confirmed by even a cursory inspection.

TWENTIETH CENTURY PRACTICE. An International Encyclopedia of Modern Medical Science. By leading authorities of Europe and America. Edited by THOMAS L. STEDMAN, M. D., New York City. In twenty volumes. Volume XIX. "Malaria and Microörganisms." New York: William Wood & Co. 1900.

This volume consists of 828 octavo pages, 520 of which are devoted to the consideration of malaria, written by Ettore Marchiafava and Amico Bignami, of the University of Rome. This article was to have been prepared for publication over a year ago, but recent discoveries regarding the malaria parasite, made even while the authors were writing the paper, necessitated delay in order that the latest knowledge pertaining to the biology of malarial parasites outside the human body might be incorporated in its pages. There is so much incompleteness in the recent literature of the subject of malaria, even much of that which has appeared within a year or two being faulty, that it is a genuine pleasure to read this work. It is so exhaustive, so elaborate, so scholarly, and withal so satisfactory that we shall not be surprised to see it quoted by teachers as one of the best and most trustworthy authority.

The section on microörganisms is written by Simon Flexner, of the University of Pennsylvania. It, also, is a comprehensive treatise, beginning with a historical introductory, then giving, in consecutive progression, the classification of microörganisms, the metabolic products of bacterial growth, the technique of bacteriology, pathogenic bacteria, and closing with a list of bibliographic references. This is a period when every physician, no mattter what his general or special line of practice, must be familiar with the general principles of bacteriology as well as with the essentials of microörganisms and their relations to medical and surgical diseases. In this monograph of 230 pages will be found just the information needed, properly classified and told in an interesting way.

The final section of the book treats of protozoa, the lowest division of the animal kingdom, and is written by Eugene L. Opie, of Johns Hopkins University. This is a most intelligent presentation of a subject that is rarely found in literature and most aptly complements the preceding sections. The text is preceded by eleven clear and handsome plates illustrating malaria, and the volume classes as one of the best of the series.

---

PROGRESSIVE MEDICINE. Volume I., 1900. A Quarterly Digest of Advances, Discoveries and Improvements in the Medical and Surgical Sciences. Edited by HOBART AMORY HARE, M. D., Professor of Therapeutics and Materia Medica in Jefferson Medical College of Philadelphia. Octavo, handsomely bound in cloth, 404 pages, 36 engravings and a colored plate. Philadelphia and New York: Lea Brothers & Co. Issued quarterly. [Price, $10.00 a year.]

This first volume of the second series of these books is presented on similar lines as were its predecessors. The same experienced editor is in charge and the list of contributors includes some of the ablest men in this country and Europe, carefully selected with reference to their fitness for the special work in hand.

The editor says, prefatorily, that every contributor has been asked to say what he has to say in a narrative form and to place his hall-mark on the text, so that it will be a story which bears a personal imprint and will express not only the views of the author cited, but the opinion of the contributor as well. It takes experience and judgment to cull such material as is worthy of attention from the great mass of literature that is thrust upon the attention of the profession. How well the work has been done one must needs examine carefully the text of each article. When this has been done the verdict must, in our judgment, be one of approval.

The articles in this number are: The surgery of the head, neck and chest, by J. Chalmers Da Costa; Infectious diseases, including acute rheumatism, croupous pneumonia, and influenza, by Frederick A. Packard; Diseases of children, by Alexander D. Blackader; pathology, by Ludwig Hektoen; laryngology and rhinology, by A. Logan Turner; and otology, by Robert L. Randolph.

A comprehensive, well-digested index closes the book.

---

A TEXT-BOOK OF DISEASES OF WOMEN. By CHARLES B. PENROSE, M. D., Ph. D., Professor of Gynecology in the University of Pennsylvania; Surgeon to the Gynecean Hospital, Philadelphia. Octavo, pp. 531. Illustrated. Third edition, revised. Philadelphia: W. B. Saunders, 925 Walnut street. 1900.

Upon the first appearance of this text-book, now some three years ago, it was pronounced by teachers and practitioners of experience to be one of the best of the modern treatises on diseases of women. That it has stood the test of time and experience and fully justified this early opinion is amply vouched for by the fact that two editions have already become exhausted. The preparation of a third edition has enabled the author to carefully revise his work and to make such additions thereto as have become necessary through increased knowledge and improved methods in gynecology.

One of the striking features of the treatise is the fact that in a large measure it is a record of the author's personal experience. Another strong point of commendation is the fact that it was written especially for the student. This has enabled the author to speak in a direct manner without circumlocution or verboseness. We may cite as an example of the author's perspicuity his chapter on Fibroid tumors of the uterus. It is rare that we have seen so much information on this subject of the right sort condensed into so little space. Another excellent reason for commending the book is the superiority of its illustrations. These are, for the most part, original, clear and self-explanatory. They are especially strong in their delineation of injuries to the perineum and of displacements of the uterus.

